# Interstitial near-infrared photoimmunotherapy: effective treatment areas and light doses needed for use with fiber optic diffusers

**DOI:** 10.18632/oncotarget.24329

**Published:** 2018-01-27

**Authors:** Shuhei Okuyama, Tadanobu Nagaya, Kazuhide Sato, Fusa Ogata, Yasuhiro Maruoka, Peter L. Choyke, Hisataka Kobayashi

**Affiliations:** ^1^ National Institutes of Health, National Cancer Institute, Molecular Imaging Program, Bethesda, Maryland 20892-1088, United States

**Keywords:** near-infrared photoimmunotherapy, interstitial light illumination, optical fiber diffuser, effective area

## Abstract

Near-infrared photoimmunotherapy (NIR-PIT), a promising cancer therapy utilizing an antibody-photoabsorber conjugate (APC) and NIR light, which induces rapid necrotic cell death only in APC-bound cells. Effective NIR-PIT in mouse models has been achieved using superficial light illumination (SLI) with light emitting diodes (LEDs) or lasers, but in the clinical setting, fiber optic diffusers have been employed to deliver light to deeper tumors. However, the performance of NIR light in tissue delivered by fiber optic diffusers is poorly understood. Here, we investigated NIR-PIT using a cylindrical fiber optic diffuser in a mouse model of A431 tumors. NIR-PIT with 100 J/cm, the same light dose used in clinical trials of NIR-PIT, was applied after insertion of the diffuser within the tumor bed, and then both bioluminescence and fluorescence imaging were analyzed to assess the therapeutic efficacy. The diffuser can deliver adequate NIR light dose for effective NIR-PIT to the A431 tumor at a distance of approximately 1 cm around the light source at 100 J/cm. At 50 J/cm NIR light effective NIR-PIT was reduced to a distance of 5 – 7 mm diameter around the light source. These results indicate that the energy of interstitial light (measured in Joules/cm) administered via a fiber diffuser determines the depth of effective NIR-PIT around the diffuser and determines the spacing at which such diffusers should be placed to entirely cover the tumor. Thermal measurements demonstrate that interstitial light for NIR-PIT does not cause damage to the skin overlying the diffuser.

## INTRODUCTION

Near-infrared photoimmunotherapy (NIR-PIT) is a promising new cancer therapy, which utilizes a monoclonal antibody (mAb)-photoabsorber conjugate (APC). Once the APC is administered systemically, it specifically binds to antigen-expressing tumor cells. Subsequent local illumination with NIR light activates the photoabsorber, IRDye700DX (IR700, silica-phthalocyanine dye), and induces cell rupture only in cells where the APC has bound. Cell killing occurs rapidly (within minutes) and results in necrotic cell death [1]. Since NIR light is totally harmless for living tissues and penetrates 1 – 2 cm into living tissues, NIR-PIT is easy-to-use for thin surface tumors. However, human tumors often project more deeply below the skin surface and thus, alternate solutions to delivering light via fiber optic diffusers are needed. In Phase 1 and 2 trials of NIR-PIT in patients with recurrent head and neck cancer where the APC targeted epidermal growth factor receptor (EGFR), interstitial fiber optic diffusers were used to treat larger tumors with good success (https://clinicaltrials.gov/ct2/show/NCT02422979).

However, little pre-clinical work has been done on interstitial fiber optic diffusers. This is because most preclinical testing is not necessary due to the small size of the animal. Regardless of how NIR light is delivered, it is clear that the light dose, light intensity, exposure time, lighting device, wavelength, etc. are critical elements in the success of NIR-PIT and these parameters have been optimized for successful NIR-PIT treatment using a superficial light application [2–4]. Successful *in vivo* NIR-PIT studies were achieved in several mouse models with subcutaneous, peritoneal, pleural, lung metastatic tumors, all illuminated externally with superficial light illumination (SLI) using light emitting diodes (LEDs) or a laser system [4–8]. SLI is an established illumination method for NIR-PIT, but is limited by the depth penetration of NIR light and potential heating of the skin. NIR light is attenuated by molecules, including water and hemoglobin, that naturally occur in living tissues and sufficient light must be delivered to overcome this barrier. In theory, NIR light can penetrate living tissues to 2.5 – 3 cm from the skin surface, but the energy of that light attenuates dramatically with distance from the skin [9] and may cause thermal injury at the skin surface. To overcome this problem, light can be administered interstitially using fiber optic diffusers embedded in catheters.

Photodynamic therapy (PDT) is a traditional phototherapy that relies on a porphyrin-based photosensitizer that produces reactive oxygen species when exposed to light ranging from visible red to NIR light. A central limitation of PDT is that it is not highly selective for tumor and off target side effects are common. However, it is clear that the ability to deliver light to a tumor is a key factor in achieving success with PDT [10]. Since tumors commonly exceed the size of light penetration in tissue, interstitial light illumination (ILI) employing an optical fiber diffuser is needed. The diffuser is inserted into a tumor through a needle or a catheter under computed tomographic or ultrasound guidance [11]. The light from the fiber optic diffuser can be delivered within the target tumor using either a front-illuminating tip, or a cylindrical light diffuser area. The latter has been typically used for PDT because it exposes a larger area. Light dose is defined as light energy per unit length using the terms J/cm, which is obtained by the product light intensity (W/cm) and exposure time (second). Typically, ILI with a laser light intensity of 100-150 mW/cm or total light dose of 100 – 300 J/cm has been applied for PDT [12]. The fibers are usually inserted at 10 - 15 mm intervals to spread light throughout tumor beds [13]. In PDT it is critical that light delivery is limited to the tumor and not overlap normal tissue due to the non-specific phototoxicity of porphyrin derivatives in normal tissue around the tumor. In contrast, light delivery in NIR-PIT does not have to be as accurate because of the high selectivity of the APC for tumor cells vs. normal tissue. Therefore, NIR light illumination can safely be administered using a cylindrical fiber optic diffuser even if the cylinder overlaps normal tissue. For clinical trials of NIR-PIT, a light dose of 100 J/cm using NIR laser light attached to a fiber diffuser has been safely used. Although such ILI has shown its clinical utility, there is little supporting data for its use in animal models.

In this study, we investigated the properties of ILI using an optical fiber diffuser for NIR-PIT in an A431 tumor-bearing mouse model. In addition, penetration of NIR light into tumors was assessed by bioluminescence imaging (BLI) and IR700 fluorescence. This information will help inform the most appropriate light doses for ILI in NIR-PIT.

## RESULTS

### ILI can deliver NIR light as well as SLI

**Figure 1:**
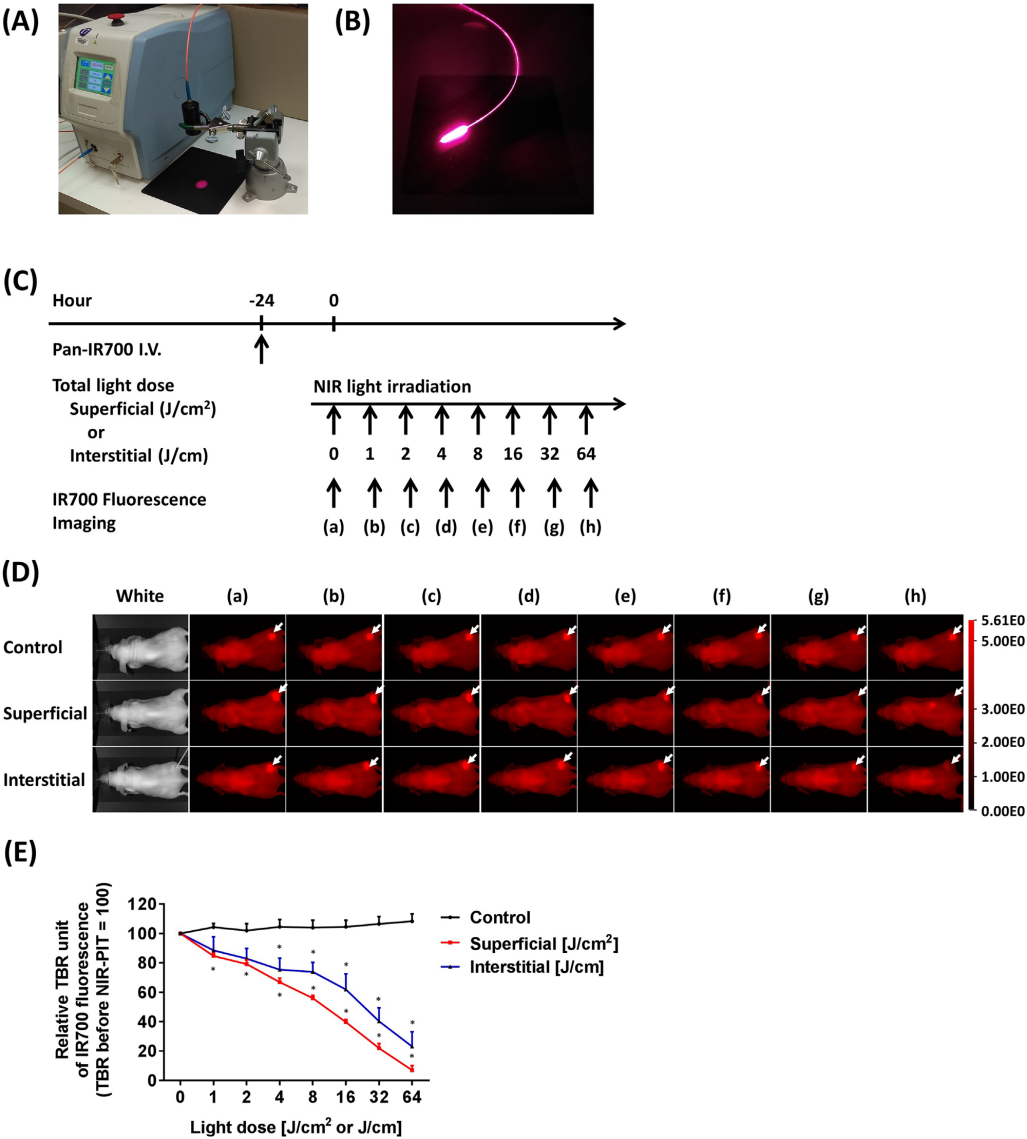
Light sources for NIR-PIT using SLI and ILI **(A)** A laser source with a collimator (black cylindrical tube) used for delivering SLI. **(B)** An optical fiber diffuser emitting 690 nm light for interstitial light delivery. **(C)** NIR-PIT regimen. IR700 fluorescence imaging was performed with progressively increasing NIR light dose as indicated. **(D)** IR700 fluorescence images were obtained at each NIR light dose by SLI and ILI. White arrows indicate IR700 accumulation within tumors. Decreased IR700 fluorescence intensity was observed in a light dose-dependent manner. **(E)** SLI significantly decreased IR700 fluorescence intensity at 1 J/cm^2^, whereas the control group showed no apparent changes. Similarly, ILI significantly decreased the intensity at 4 J/cm (*n* = 5; ^*^, *P* < 0.05 vs. control).

To monitor the effects of fiber-diffuser-administered light on IR700 fluorescence intensity compared to SLI, we performed NIR-PIT in tumors embedded just under the mouse skin using both ILI and SLI (Figure 1A, 1B). IR700 fluorescence images were obtained at each light dose (Figure 1C). NIR light illumination resulted in decreased IR700 fluorescence in a light dose dependent fashion due to photobleaching, whereas no apparent changes in IR700 fluorescence were shown in the control group (Figure 1D). Compared to the control group, SLI significantly decreased IR700 fluorescence intensity at 1 J/cm^2^, whereas ILI showed a significant decrease at 4 J/cm (each *n* = 5) (Figure 1E). The variation of IR700 fluorescence with increasing energy with ILI was wider than that with SLI. This is likely because light delivery via the fiber diffuser is strongly dependent on precise geometrical relationships between the insertion site and the tumor and is therefore, subject to more variability. However, both SLI and ILI resulted in decreased IR700 fluorescence to an average of only 20% or less of the original intensity. Thus, these results indicated that ILI delivered via a fiber diffuser delivered NIR light in a manner comparable to SLI.

### Thermal effects induced by ILI

**Figure 2:**
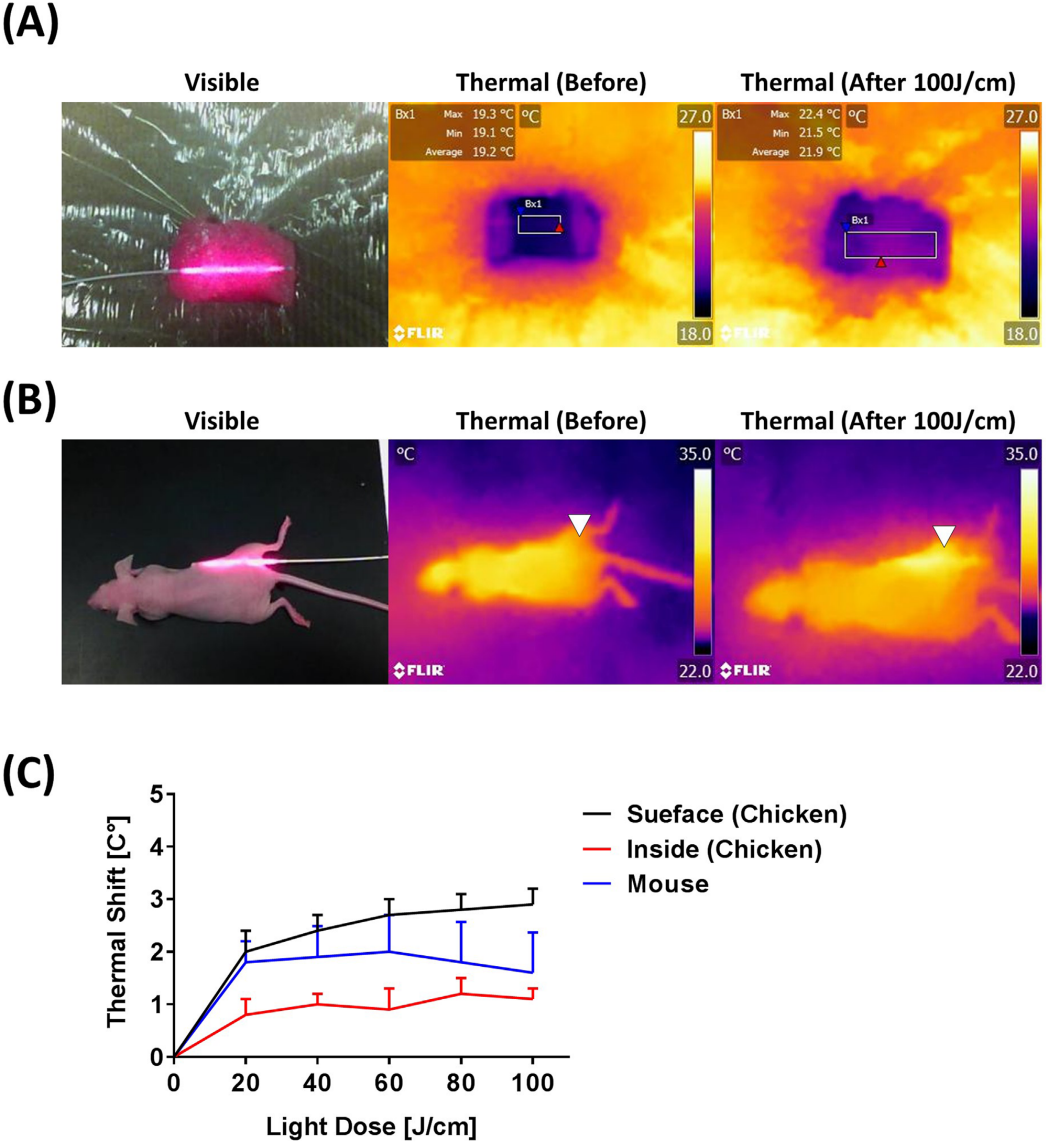
Thermal effects seen with an optical fiber diffuser **(A)** Visible and thermal images to detect thermal effects induced by the 100 J/cm ILI in chicken breast. **(B)** Visible and thermal images obtained before and after NIR light illumination at 100 J/cm. White triangles indicate the diffuser insertion area. **(C)** Temperature shifts induced by NIR light exposure show minor thermal shifts. For instance, ILI produces only minimal temperature changes.

Thermal effects during 100 J/cm ILI were observed at about 330 mW/cm intensity. Thermal effects were measured at several sites (Figure 2A and 2B), but only slight thermal shifts of 1 - 2 degrees C were observed, indicating that ILI had little or no thermal adverse effects in live animals (Figure 2C).

### Estimation of ILI NIR-PIT efficacy

**Figure 3:**
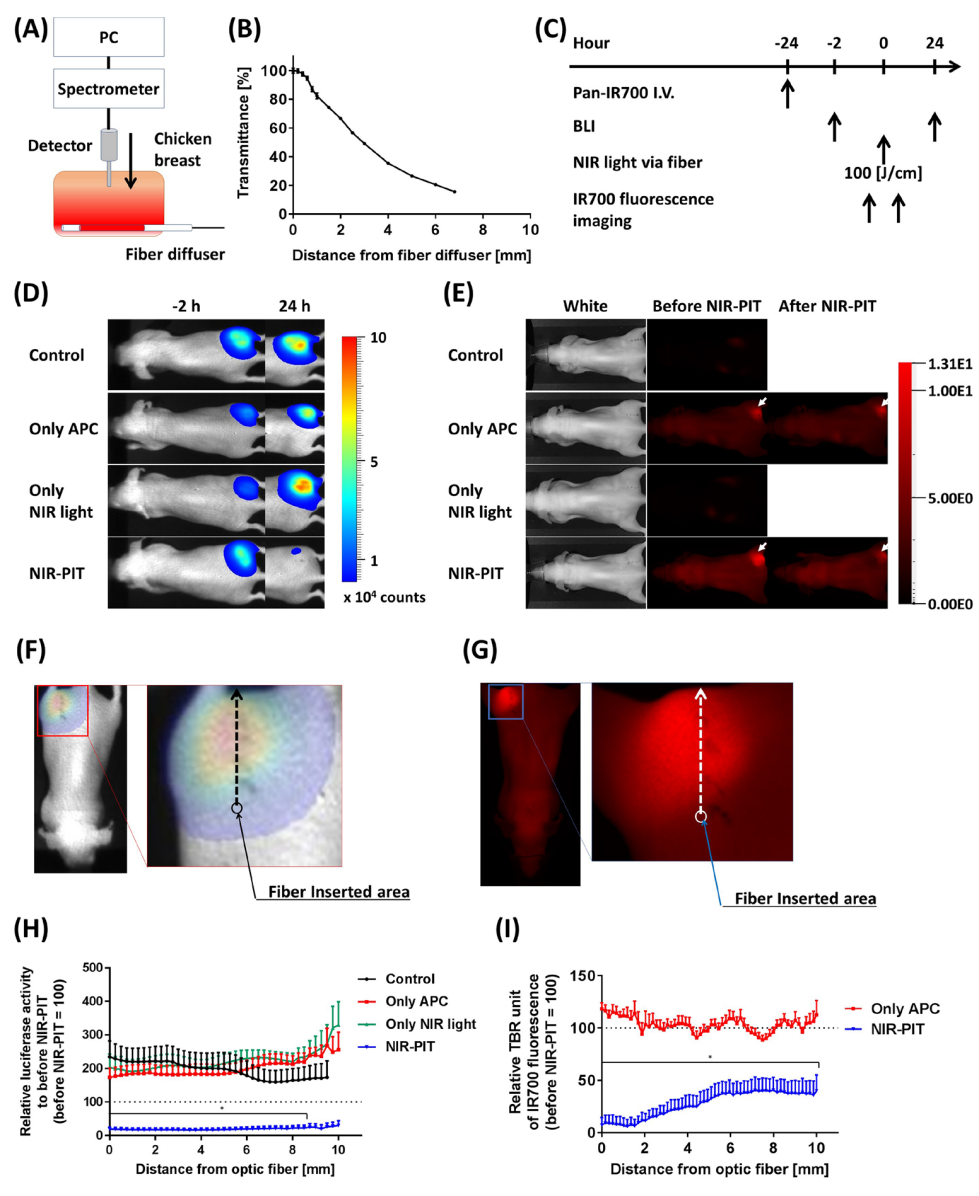
Estimation of effective NIR-PIT area using the fiber diffuser **(A)** Schema of NIR light transmittance measurement using chicken breast. **(B)** NIR light transmittance at chicken breast as a function of distance from the fiber diffuser. **(C)** NIR-PIT regimen. Both BLI and IR700 fluorescence imaging were performed according to this regimen. **(D)** Bioluminescence images before (-2 hours) and at 1 day after (24 hours) NIR-PIT. Although tumor sizes among each group were approximately the same after NIR-PIT, only the NIR-PIT group mice showed a dramatic decrease in BLI at 24 hours after NIR-PIT. **(E)** IR700 fluorescence images before and immediately after NIR-PIT. White arrows indicate IR700 accumulation in tumors. Control and Only NIR light groups, exhibited no IR700 fluorescence. The fluorescence decreased in the NIR-PIT-treated mice, indicating NIR light was delivered and resulted in photobleaching of IR700. **(F, G)** Schemas of NIR-PIT effective area analysis from both BLI and IR700 fluorescence image. The light intensities at each pixel were analyzed as indicated by the arrows, and then compared to before NIR-PIT. **(H)** Relative luciferase activity compared to before NIR-PIT at each distance from the light source. NIR-PIT group exhibited dramatically decreasing luciferase activity, and significantly differences were seen between 0 – 8.5 mm distance from the light source (*n* = at least 7; ^*^, *P* < 0.05 of NIR-PIT group vs. the other groups). **(I)** Relative IR700 fluorescence ratio comparing before and after NIR-PIT. NIR-PIT group showed significantly decreased IR700 fluorescence intensity within a 10 mm area from fiber diffuser (*n* = at least 7; ^*^, *P* < 0.05 vs. Only APC).

In order to estimate the effective area of NIR-PIT when using a fiber optic diffuser, we first measured typical NIR light transmittance in living tissue using a chicken breast. A light detector, attached to a computer, was vertically inserted into the sample at right angles to the fiber optic diffuser (Figure 3A). The transmittance of light was highest near the diffuser and decreased as a function of distance reaching approximately 20% at a distance of 7 mm (Figure 3B). BLI and IR700 fluorescence before and after 100 J/cm interstitial NIR-PIT were also performed (Figure 3C). The NIR-PIT treated group exhibited a decrease in BLI at 24 hours while IR700 fluorescence intensity decreased immediately after NIR-PIT (Figure 3D, 3E). The NIR-PIT effective area was determined based on pixel-by-pixel analysis of both BLI and IR700 fluorescence intensity (Figure 3F, 3G). In comparison to the control group, NIR-PIT-treated mice exhibited significantly decreased light intensity on BLI at a distance of 8.5 mm distance from the light source (Figure 3H). Similarly, IR700 fluorescence significantly decreased up to at least a distance of 10 mm from the light source (Figure 3I). These results indicated that ILI at 100 J/cm delivered adequate NIR light for NIR-PIT at approximately 8.5 – 10 mm distance from the fiber optic diffuser.

### Fiber optic delivered interstitial NIR light dose is sufficient for NIR-PIT

**Figure 4:**
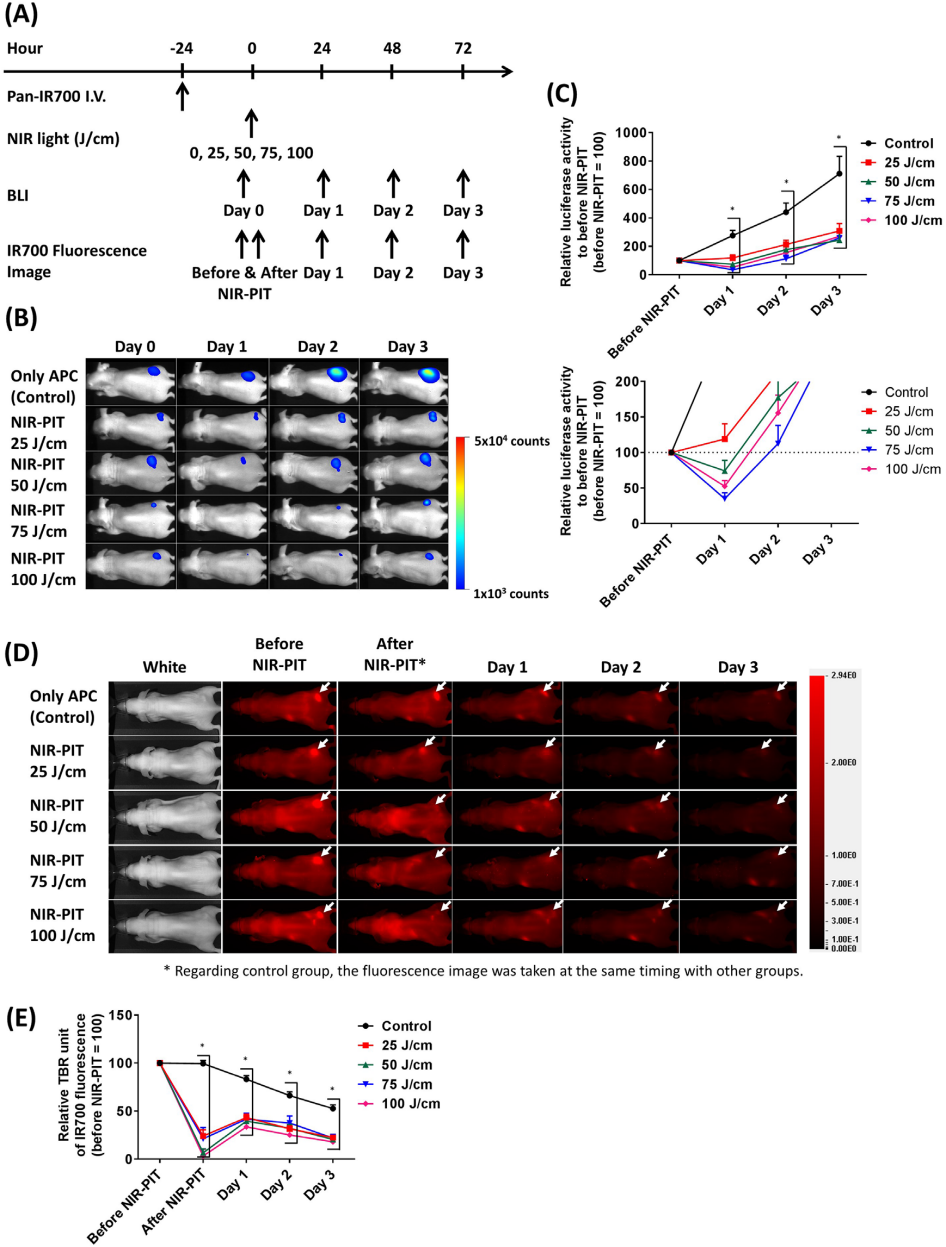
Estimation of interstitial NIR light dose needed for successful NIR-PIT treatment **(A)** NIR-PIT regimen. Both bioluminescence and IR700 fluorescence images were obtained at each time point as indicated. **(B)** Bioluminescence images in response to NIR light dose. The control group gradually increased luciferase activity due to tumor growth, but NIR-PIT groups exhibited marked decrease in BLI. **(C)** BLI before and after NIR-PIT. Although NIR-PIT groups showed significantly differences with the control group, NIR light more than 50 J/cm were needed to significantly decrease the activity compared to before NIR-PIT. However, all activities were increased at 2 days after treatment (*n* = at least 7; ^*^, *P* < 0.05 vs. control). **(D)** IR700 fluorescence images of control and NIR-PIT groups. White arrows indicate tumors. Although IR700 fluorescence was strongly decreased after 25 J/cm or more NIR light irradiation only modest changes in tumor were seen with only 25 J/cm. **(E)** IR700 fluorescence intensities were significantly decreased immediately after NIR-PIT with 25 J/cm or more NIR light dose (*n* = at least 7; ^*^, *P* < 0.05 vs. control). Although the intensities increased at 1 day after NIR-PIT, they afterward decreased as well as the control group.

To determine how much NIR light dose is typically required for achieving cell killing with NIR-PIT, we carried out NIR-PIT in A431 tumor-bearing mice with several interstitially administered NIR light doses (Figure 4A). At first, BLI light intensity of tumors was measured before and at 1 day, 2 days, and 3 days after NIR-PIT. BLI decreased at 1 day after NIR-PIT when 50 J/cm or more NIR light was administered. However, after this initial decrease, increasing BLI light intensity were seen in the tumors at later times due to rapid tumor regrowth in this aggressive tumor model (Figure 4B, 4C). IR700 fluorescence intensities were also observed at similar time points as BLI. All NIR-PIT-treated groups showed significantly decreased IR700 fluorescence after NIR-PIT whereas the control group retained its IR700 fluorescence. Although all NIR-PIT-treated mice exhibited increased IR700 fluorescence at 1 day after NIR-PIT (due to refreshing of circulating panitumumab - IR700 conjugate (Pan-IR700) into the tumor) the signal continued to decrease 2 days after NIR-PIT as well as in the control group (Figure 4D, 4E). These results suggested that 25 J/cm ILI was not enough for successful NIR-PIT, but 50 J/cm or more NIR light can successfully result in cell killing at a distance of 5 - 7 mm size from the fiber optic diffuser.

## DISCUSSION

In this study, we demonstrated that an optical fiber diffuser can deliver sufficient NIR light to kill cancer cells within a cylinder shaped region around the diffuser extending at least 5 - 7 mm from the diffuser surface. It is well known that NIR light is absorbed by molecules such as hemoglobin, melanin and water, resulting in insufficient numbers of photons available for effective NIR-PIT in deep or bulky tissues [14]. To overcome this limitation, the fiber diffuser has been used to introduce light within the interstitium of the tumor. Compared with SLI using LEDs or lasers, such diffusers can be inserted within the tumor and deliver NIR light directly to the targeted area, enabling treatment of larger and deeper tumors than is possible with SLI alone [15, 16]. The penetration depth of 650 - 900 nm wavelength light, which is defined as the depth at the area where the collimated incident light intensity decreases to 1 / e (approximately 37 %) [17], is reported to be 2 - 3 mm for human or mouse skin tissues [18–20], 4 mm for human mucous tissues [20] and 3 - 4 mm for rat muscle tissues calculated from the reference data [20]. If these penetration depths are simply applied to the mouse subcutaneous tumor model in this study, the superficial light intensity should decrease by approximately 60 - 70% when it goes through about 1 mm mouse skin (${\left(\frac{1}{e}\right)}^{1/2}≅0.6,{\left(\frac{1}{e}\right)}^{1/3}≅0.72$). This decayed light additionally decreases when it penetrates into the subcutaneous tissues where it is scattered by adipocytes, thus only a few percent of incident light reaches tissues 15 mm deep area from the skin surface due to light absorbance and scattering. Typically, *in vivo* NIR-PIT studies have been conducted with SLI using 50 - 100 J/cm^2^ NIR light, which attenuates to below 1 J/cm^2^ at nearly 15 mm depth. Notwithstanding these theoretical calculations A431 tumor cells treated with only 0.5 J/cm^2^ NIR light showed cell death [21]. Thus, even this low NIR light dose was sufficient for NIR-PIT although higher light doses were preferred. It is likely that scattered and reflected light in the tissue also contributed to the effective light dose of NIR-PIT. Interestingly, Welch et al. demonstrated that light scattering can create more light just inside the tissues than direct irradiance [17]. This may explain why NIR-PIT is effective even at a depth of 15 mm with 50 -100 J/cm^2^. However, beyond this point the effectiveness of NIR-PIT rapidly drops off and tumors that extend deeper will require ILI. ILI using the fiber diffuser has no limitation regarding depth because it can deliver light homogeneously along the axial direction of the fiber. Of course, the light dose attenuates as it goes through tissues around the light source, but sufficient NIR light dose can be delivered for NIR-PIT to a distance of 10 mm when using 100 J/cm NIR light. This result indicates that the fiber diffusers should be inserted at intervals of 15 - 20 mm interval to completely cover the tumor to be treated.

Only minor thermal effects could be seen at the skin surface with thermal cameras during ILI. The thermal shift with 100 J/cm NIR light irradiation at approximately 330 mW/cm using the fiber diffuser was negligible and does not result in thermal injury. With SLI, the tissue can heat up if more energy is delivered to the skin surface. This can be ameliorated simply by moving air over the surface as with a simple ventilation fan [22]. In contrast, ILI can deliver sufficient light dose without raising the surface skin temperature.

We also investigated the NIR light threshold required for NIR-PIT via the fiber diffuser. In this study, the fiber was inserted adjacent to the tumor and 25 -100 J/cm NIR light was delivered. Although 25 J/cm NIR light showed only mild killing effects, it was not enough for complete tumor killing. Based on placing the fiber diffuser in chicken, we found that roughly 20 % of the incident light could be measured at a distance of 7 mm from the fiber, (Figure 3B), it was clear that 25 J/cm resulted in insufficient light intensities at depth. Increasing the light dose to at least 50 J/cm seems to overcome this limitation. An alternative to increasing the light intensity is to perform repeated NIR-PIT at a lower light intensity [2, 23], a technique we will examine in future studies.

To clarify the pre-clinical effect that would be relevant to clinical outcome, superior tumor models such as surgically orthotopic implantation tumor models are better than subcutaneous xenografted tumor models [24–26], yet surgical orthotopic implant requires highly trained surgical skills. Therefore, in this proof-of-principle study of NIR-PIT with interstitial NIR light exposure using an optical fiber diffuser, we chose a simple subcutaneous xenograft tumor model. For monitoring therapeutic effects of cancers, fluorescent proteins transfected to tumor cells are a powerful imaging method because of the brightness compared with luciferase photon-counting which was used in this study [27–29]. However, luciferase photon-counting evaluated acute therapeutic effects of NIR-PIT better than fluorescence imaging with green fluorescent protein [30]. Therefore, in this study, we employed luciferase photon-counting for reading out tumor cell killing.

In conclusion, ILI using an optical fiber diffuser was used to treat tumors with NIR-PIT in A431 tumors. At 100 J/cm tumors were killed to a distance of around 10 mm from the light source while at 50 J/cm cells were killed at 5 -7 mm from the light source. Neither dose resulted in thermal toxicity. These findings could inform the application of optical fiber diffusers when applied to clinical NIR-PIT.

## MATERIALS AND METHODS

### Reagents

Water soluble, silica-phthalocyanine derivative, IRDye700DX NHS ester (IR700; C_74_H_96_N_12_Na_4_O_27_S_6_Si_3_, molecular weight of 1954.22) was obtained from LI-COR Bioscience (Lincoln, NE). Panitumumab, a fully humanized IgG2 mAb directed against EGFR, was purchased from Amgen (Thousand Oaks, CA). All other chemicals were of reagent grade.

### Synthesis of IR700-conjugated panitumumab

Conjugation of dyes with mAb has been previously described [31]. Briefly, panitumumab (1 mg, 6.8 nmol) was incubated with IR700 (66.8 μg, 34.2 nmol, 5 mmol/l in dimethyl sulfoxide (DMSO)) in 0.1 mol/l Na_2_HPO_4_ (pH 8.5) at room temperature for 1 h. Subsequently, the mixture was purified with a Sephadex G25 column (PD-10; GE Healthcare, Piscataway, NJ). The protein concentration was determined with a Coomassie Plus protein assay kit (Pierce Biotechnology, Rockford, IL) by measuring the absorption at 595 nm (8453 Value System; Agilent Technologies, Santa Clara, CA). The concentration of IR700 was measured by its absorption to confirm the number of fluorophore molecules conjugated to each mAb.

### Cell lines and culture

A431-luc cells stably expressing EGFR and luciferase were grown in RPMI1640 supplemented with 10 % fetal bovine serum and 1 % penicillin-streptomycin in tissue culture flasks in a humidified incubator at 37 °C in an atmosphere of 95 % air and 5 % carbon dioxide.

### Animal and tumor models

All *in vivo* procedures were conducted in compliance with the Guide for the Care and Use of Laboratory Animal Resources (1996), US National Research Council, and approved by the local Animal Care and Use Committee. Six- to eight-week-old female homozygote athymic nude mice were purchased from Charles River (Frederick, MD). During the procedure, mice were anesthetized with isoflurane. To evaluate NIR-PIT effect using a fiber optic diffuser in the mouse tumor model, two million of A431-luc cells in 200 μL phosphate-buffered saline (PBS) was administered just below the skin of their right dorsa. The mice were used for experiments 7 - 10 days after cell inoculation.

### NIR-PIT treatment

A 0.98 mm diameter cylindrical fiber optic diffuser with 30 mm irradiation length was purchased from Medlight SA (Ecublens, Switzerland). To check whether the diffuser can deliver NIR light to tumor beds, we inserted the diffuser just next to the tumors on the right dorsum of the mice which had previously received 100 μg of Pan-IR700 i.v. at 24 hours prior to NIR-PIT treatment. Thereafter, we observed IR700 fluorescence within the tumor using methods detailed below. Light dose was escalated in a step wise manner from 0, 1, 2, 4, 8, 16, 32 to 64 J/cm by exposing consistent light power at 330 mW/cm. At the same time, mice in the control group were also observed. Prior to placing the diffuser into the tumor, an 18G needle with a translucent catheter (SR-OX1864CA; TERUMO, Tokyo, Japan) was inserted vertically. The catheter transmitted almost all light at NIR wavelengths used in this study (its transmittance is approximately 97%). Afterward, the trocar was removed and the diffuser was inserted carefully into the catheter leaving it within the tumor. A photobleaching effect on IR700 occurs after NIR light illumination whereby successfully exposed tumor no longer fluoresce. This was observed initially with SLI using a NIR laser (BWF5-690-8-600-0.37; B&W TEK INC., DE) with a collimator (COL-SMA-BK7-12.7mm; B&W TEK INC.) at NIR light doses of 0, 1, 2, 4, 8, 16, 32 and 64 J/cm^2^ by exposing consistent light power at 300 mW/cm^2^. The light intensities were measured by an integrating sphere sensor (S142C; Thorlabs, Newton, NJ) attached to a power meter (PM100D; Thorlabs). Exposure times were determined based on the measured light intensity.

To evaluate how far the diffuser could deliver NIR light into living tissue, we inserted the diffuser into commercially obtained chicken breast and investigated light transmittance as a function of distance from the light source. Thereafter, for investigating NIR-PIT efficacy using the diffuser, A431 tumor-bearing mice were divided randomly into 4 groups with at least 7 animals in each group: (1) no Pan-IR700, no NIR light exposure but the diffuser inserted near tumor (control); (2) 100 μg of Pan-IR700 i.v., no NIR light exposure but the diffuser inserted (only APC); (3) NIR light exposure only, NIR light was administered at 100 J/cm via the diffuser (only NIR light); (4) 100 μg of Pan-IR700 i.v., followed by NIR light exposure at 100 J/cm via the diffuser (NIR-PIT). APCs were administered intravenously to the mice 24 hours prior to performing NIR-PIT. To maintain the mouse position between imaging procedures tiny dots were applied to the mouse’s skin with an indelible marker. The diffuser was inserted at one of the marked places and the other marked places were used as guides. The mice within each group were observed for both luciferase activity and IR700 fluorescence before and after NIR-PIT treatment. These experiments were performed when the tumor reached a size of approximately 10 mm.

To estimate how much light dose is required to achieve NIR-PIT with the diffuser, A431 tumor-bearing mice were randomly divided into 5 groups with at least 10 animals for the following treatment: (1) 100 μg of Pan-IR700 with no NIR light illumination but the diffuser inserted (control); (2-5) 100 μg of Pan-IR700 with 25, 50, 75 or 100 J/cm NIR light illumination via the diffuser, respectively. Pan-IR700 was intravenously injected to mice through the tail vein 24 hours prior to NIR light illumination. Both images of luciferase activity and IR700 fluorescence were taken just before and at 24, 48 and 72 hours after NIR-PIT.

### IR700 fluorescence imaging study

IR700 fluorescence images were obtained from a Pearl Imager (LI-COR Bioscience). Such images were obtained with a 700-nm fluorescence channel and regions of interest (ROIs) were manually drawn on both the right dorsum (tumor) and the left dorsum (background). The average fluorescence intensities of each ROIs were measured by Pearl Cam Software (LI-COR Bioscience), then the tumor-to-background ratio (TBR) of fluorescence intensities were calculated according to a previous report [22].

To determine effective light delivery with a fiber optic diffuser, fluorescence intensities at each pixel (170 μm spatial resolution) were analyzed from the place of diffuser insertion, to another site approximately 10 mm distant. Subsequently, the intensities during NIR-light exposure were divided by those before NIR-light exposure to calculate relative fluorescence intensity.

### BLI study

For BLI, D-luciferin (15 mg/ml, 200 μg) was injected intraperitoneally into the mice, and imaging was performed 5 minutes later (PhotonIMAGER; Biospace Lab). Light intensity per unit area and time were quantified by placing ROIs on the tumor implanted on the right dorsum and a background ROIs over the corresponding left dorsum. TBR was calculated before and after NIR-PIT. To estimate the effective area of NIR-PIT, light intensity was measured on a pixel-by-pixel basis and then, compared to background. To determine the effective area of NIR-PIT, light intensities at each pixel (250 μm spatial resolution) were analyzed in the same way as the IR700 fluorescence imaging study.

### Thermal imaging study

To estimate thermal effects after ILI using a fiber diffuser, we obtained thermal images using a thermal imaging system (FLIR C2, FLIR Systems, Inc., Wilsonville, OR) while the diffuser delivering 100 J/cm NIR light. Skin surface air cooling was provided by a consumer-grade desktop fan (Holmes, Boca Raton, FL) under the following situations: (1) the diffuser was placed within the chicken breast; (2) the diffuser was horizontally inserted superficially (about 1 mm depth from the top surface) of the chicken breast; (3) the diffuser was horizontally inserted under the skin of a live anesthetized mouse. These images were obtained at a total NIR light dose of 0, 20, 40, 60, 80 and 100 J/cm. Analysis of thermal images has been described previously [22].

### Statistical analysis

Data were expressed as mean ± standard error. Statistical analyses were carried out using GraphPad Prism version 7 (GraphPad Prism; GraphPad Software Inc., La Jolla, CA). For multiple comparisons, a one-way analysis of variance (ANOVA) followed by the Bonferroni correction for multiple comparisons was used. Student’s *t* test was also used for comparison between two groups. *P*-values of less than 0.05 were considered statistically significant.

## Abbreviations

NIR-PIT: near infrared Photoimmunotherapy
NIR: near infrared
APC: antibody-photoabsorber conjugate
mAb: monoclonal antibody
EGFR: epidermal growth factor receptor
FDA: Food and Drug Administration
ROI: region of interest
LED: light emitting diode
TBR: tumor-to-background ratio
DMSO: dimethyl sulfoxide
ANOVA: one-way analysis of variance
SLI: superficial light illumination
ILI: internal light illumination
PDT: photodynamic therapy
PBS: phosphate-buffered saline
BLI: bioluminescence imaging
